# Genetic Dissection of Growth Traits and Identification of Candidate Genes in Japanese Flounder (*Paralichthys olivaceus*) Using Single- and Multi-Trait Genome-Wide Association Analyses

**DOI:** 10.3390/biology15151246

**Published:** 2026-07-28

**Authors:** Shuo Liang, Ao Chen, Zhili Tuo, Jianhua Xu, Yong Chen, Li Jiang

**Affiliations:** 1 National Demonstration Center for Experimental Fisheries Science Education, Shanghai Ocean University, Shanghai 201306, China; 13722204240@163.com (S.L.); m19503860141@163.com (A.C.); 2 Research Centre for Aquatic Biotechnology, Chinese Academy of Fishery Sciences, Beijing 100141, China; 3 Beijing Key Laboratory of Fishery Biotechnology, Chinese Academy of Fishery Sciences, Beijing 100141, China; 4 Key Laboratory of Aquatic Genomics, Ministry of Agriculture and Rural Affairs, Beijing 100125, China; 5 Laiyuan Aquatic Products Station, Laiyuan County Agriculture and Rural Affairs Bureau, Baoding 074399, China; 6 Baotou Tianyou Ecological Agricultural Technology Co., Ltd., Baotou 014060, China

**Keywords:** Japanese flounder (*Paralichthys olivaceus*), genome-wide association study, growth traits, candidate genes, KEGG pathway, pleiotropic loci

## Abstract

Genetic improvement of growth traits is a key objective in aquaculture breeding. In this study, we performed whole-genome resequencing of 282 Japanese flounder individuals from a single farmed cohort and obtained approximately 6.67 million high-quality SNPs. Single-trait and multi-trait GWAS were conducted for 11 growth traits: body weight (BW), total length (TL), body length (BL), body depth (BD), trunk length (TUL), head length (HL), snout length (SnL), caudal peduncle depth (CPD), eye diameter (ED), postorbital head length (PoL), and interorbital width (IW). Heritability estimates ranged from 0.172 (IW) to 0.487 (HL). Head length showed the highest heritability, followed by PoL (0.328), ED (0.323), BD (0.300), and CPD (0.291). Genetic correlation analysis revealed that BW was strongly positively correlated with most body size traits (*r_g_* > 0.9), indicating that simultaneous improvement of these traits is feasible. Single-trait GWAS identified a total of 55 suggestive associated loci (top five SNPs per trait, combined across all 11 traits, at *p* < 1 × 10^−5^). Notably, we observed pronounced pleiotropic effects, where a single SNP or gene influences multiple growth traits. For example, multiple SNPs on chromosome 14 were associated with more than five traits (TL, BL, BD, TUL, BW), and the *fras1* gene affected both head length and post-orbital head length, demonstrating a genetic basis of pleiotropy. Additionally, *fras1* was associated with head length, and *nkd1* with interorbital width. Multi-trait GWAS uncovered 12 novel loci not detected in single-trait analyses, with candidate gene annotation pointing to pathways such as ECM–receptor interaction (e.g., *fras1*, *lamb4*) and Wnt signaling (e.g., *nkd1*), suggesting that these biological processes may underlie the observed associations. These findings are preliminary and require validation in larger independent cohorts. KEGG enrichment analysis showed significant enrichment in ECM–receptor interaction and non-homologous end-joining pathways (corrected *p* < 0.05). Our results provide a genetic basis for growth trait improvement and candidate markers for breeding programs in Japanese flounder.

## Simple Summary

Growth traits are economically important in Japanese flounder aquaculture, but the genetic architecture underlying these complex traits remains incompletely understood. In this study, we performed whole-genome resequencing of 282 Japanese flounder individuals and conducted both single-trait and multi-trait genome-wide association studies (GWAS) for 11 growth traits. We estimated heritability values ranging from 0.172 to 0.487 and found strong positive genetic correlations among most body size traits. We identified 55 significant loci via single-trait GWAS and uncovered 12 additional novel loci using multi-trait analysis, demonstrating the value of multi-trait approaches for detecting pleiotropic genes. Key candidate genes included fras1 (associated with head length) and *nkd1* (associated with interorbital width). KEGG enrichment analysis revealed significant enrichment in the ECM–receptor interaction and non-homologous end-joining pathways. Our findings provide important insights into the genetic basis of growth traits and offer candidate markers for marker-assisted selection in Japanese flounder breeding programs.

## 1. Introduction

Global fish consumption has grown faster than that of any other animal protein source for over 60 years. Aquaculture currently contributes 46% of total fish production and approximately half of the fish directly consumed by humans (FAO, 2020) [1]. China is the world’s leading fish producer. According to the 2024 National Fishery Economic Statistics Bulletin (Ministry of Agriculture and Rural Affairs of China, Bureau of Fisheries, 2025), the country’s total aquatic production reached 73.5759 million tons in 2024, of which 60.6003 million tons came from aquaculture, accounting for approximately 60% of global aquaculture output [2]. Selective breeding has traditionally aimed to improve production efficiency of economically important traits. In recent years, the incorporation of genomic marker information—i.e., genomic prediction or marker-assisted selection (MAS)—has been shown to increase selection accuracy and accelerate genetic gain [3].

Growth traits are directly linked to production and profit, making them primary targets for aquaculture breeding programs. Although growth is a polygenic quantitative trait, the use of genomic information can accelerate genetic progress compared to phenotypic selection alone [4]. Therefore, despite the relatively high cost of initial genotyping, gene-environment interaction breeding programs based on genomic information have been widely adopted and have achieved success in species such as ayu (*Plecoglossus altivelis*) [5], Atlantic salmon [6,7], Senegalese sole [8], Pacific white shrimp [9], and common carp [10].

Japanese flounder (*Paralichthys olivaceus*), also known as olive flounder, belongs to the class Actinopterygii, order Pleuronectiformes, family Paralichthyidae. It is a prime candidate for aquaculture due to its fast growth, tolerance to temperature fluctuations, and high feed conversion efficiency, making it one of the most economically important species in the region [11,12]. Under normal farming conditions, it takes about 15 months to reach market weight (1–2 kg). Previous studies have estimated the heritability of growth traits such as body weight and body length to range from 0.12 to 0.48 [13,14,15,16], indicating that these traits are under moderate genetic control and thus amenable to genetic improvement through selective breeding. In Japanese flounder, a GWAS for growth traits was recently conducted by Omeka et al. (2022) [11], who identified several QTLs for body weight and length. However, that study focused primarily on single-trait analyses and did not explore the genetic correlations among multiple traits or the potential of multi-trait GWAS to uncover pleiotropic loci. Furthermore, the genetic architecture of head-related traits (such as head length, snout length, and interorbital width) remains largely unexplored in this species.

GWAS is a powerful approach for identifying genetic variants associated with complex traits by analyzing dense genotyping data from large populations [17,18]. Based on linkage disequilibrium (LD), GWAS can infer potential trait-associated loci [19,20]. In aquaculture, GWAS has been successfully applied to locate genomic regions and candidate genes for growth, disease resistance, and stress tolerance in species including Japanese flounder [21], large yellow croaker [5], rainbow trout [22], Atlantic salmon [23], common carp [5], and ayu [24].

Despite these previous findings, the genetic architecture underlying the complex interrelationships among multiple growth traits in Japanese flounder remains poorly understood. In particular, the extent of pleiotropy (i.e., shared genetic control among traits) and the utility of multi-trait GWAS for uncovering novel loci have not been systematically explored in this species. Therefore, the objectives of this study were: (1) to estimate genetic parameters (heritability and genetic correlations) for 11 growth traits; (2) to identify trait-associated loci via both single-trait and multi-trait GWAS; and (3) to annotate candidate genes and pathways that may regulate growth, with the ultimate goal of providing a foundation for marker-assisted selection in Japanese flounder breeding programs.

## 2. Materials and Methods

### 2.1. Experimental Population and Phenotypic Measurement

A total of 400 individuals from the same cohort were collected from a farm in Yangjiapo Town, Binhai New Area, Tianjin, China. Based on available pedigree records, we selected individuals that were as unrelated as possible (avoiding full- and half-sib matings) to minimize population structure effects. All phenotypic measurements were performed following a standardized protocol established in our laboratory. Body weight (BW) was measured using an electronic balance (Shanghai Jingtian Electronic Instrument Co., Ltd., Shanghai, China; precision: 0.01 g). Total length (TL), body length (BL), body depth (BD), trunk length (TUL), head length (HL), snout length (SnL), caudal peduncle depth (CPD), eye diameter (ED), postorbital head length (PoL), and interorbital width (IW) were measured using digital calipers (Guilin Guanglu Measuring Instrument Co., Ltd., Guilin, China; precision: 0.01 mm). Before collection, fish were euthanized by immersion in a buffered tricaine methanesulfonate (MS-222, 150 mg/L) solution, and then tail fin tissue was sampled for DNA extraction.

### 2.2. DNA Extraction, Sequencing and Genotyping

Total DNA was extracted from caudal fin tissue using the phenol-chloroform method. Integrity was assessed by agarose gel electrophoresis, and concentration was measured with a fluorometer. Qualified samples were subjected to paired-end (PE150) sequencing on the DNBSEQ platform at BGI. Raw reads were filtered with SOAPnuke(v2.1.9), and genotyping was performed using GATK (v4.1.8.0) (HaplotypeCaller + GenotypeGVCFs). Variants were filtered with BCFtools (1.23.1) (DP < 10, MAF < 0.01), and missing genotypes were imputed with BEAGLE (v4.1). PLINK (v1.9) quality control removed individuals with missing genotype rate > 5%, SNPs with missing rate > 5%, MAF < 0.01, and those significantly deviating from Hardy–Weinberg equilibrium (HWE, $p<1\times 10^{-6}$). The final dataset comprised 282 individuals and 6,668,023 high-quality SNPs. Of the 400 sampled fish, 282 passed all quality control filters (individual missing genotype rate > 5%, SNP missing rate > 5%, MAF < 0.01, and Hardy–Weinberg equilibrium deviation at *p* < 1 × 10^−6^) and were retained for final analyses.

### 2.3. Population Genetics and Genetic Parameter Estimation

PLINK (v1.9) was used for PCA of the population structure. The indices of genetic diversity, including observed heterozygosity (Ho), expected heterozygosity (He) and the inbreeding coefficient (F), were calculated with VCFtools (v0.1.17) and PLINK (v1.9).

GCTA (v1.94.1) [25] was used to construct a genomic relationship matrix (GRM) using the formula G = (M − P)(M − P)^T^/[2Σp_i_(1 − p_i_)], where M is the genotype matrix and P is the allele frequency matrix.

Heritability for each trait was estimated using a univariate linear mixed model: y = Xb + Za + e, where y is the phenotype vector, b is the vector of fixed effects (age, sex, and the first three PCs), a is the vector of additive genetic effects assumed ~N(0, Gσ^2^_a_) with G being the GRM, and e is the residual error. Variance components were estimated using restricted maximum likelihood (REML). Coefficients of variation (CV) were calculated as (SD/mean) × 100% for each trait.

Genetic correlations between traits were estimated using bivariate linear mixed models implemented in GCTA, with the same fixed effects as in the univariate models. Standard errors of the correlations were obtained by the delta method.

### 2.4. Genome-Wide Association Study

Single-trait GWAS was performed using three software packages: GEMMA (v0.98.5) [25], the R package BOLT-LMM, and GmatS (v1.01). Age, sex, and the first three principal components (PC1–PC3) were included as covariates to account for population stratification. Multi-trait GWAS was carried out on the four trait combinations (grouped based on functional and morphological relevance: (1) TL + BL + BW; (2) BL + BD; (3) HL + SnL + PoL; (4) ED + IW + CPD) using GEMMA_mv, the R package data.table, and GmatS_mv.

To balance the detection of true associations with the risk of false positives, we adopted a dual-threshold strategy. The genome-wide suggestive significance threshold was set at *p* < 1 × 10^−5^ to avoid missing potentially important loci. In addition, a more stringent genome-wide significant threshold was determined by Bonferroni correction as *p* < 7.5 × 10^−9^ (0.05/6,668,023) to control for multiple testing [17]. The genomic inflation factor (λ) was calculated for each GWAS to assess potential population stratification. Values close to 1.0 indicate adequate correction for population structure. Manhattan plots [26] and Q-Q plots [27] were generated using R (v4.5.2).

### 2.5. Annotation of Candidate Genes and Functional Enrichment Analysis

For each trait, the top five SNPs were extended 50 kb upstream and downstream to define candidate regions [28]. Candidate genes were extracted from the Japanese flounder (*Paralichthys olivaceus*) reference genome (GCF_024713975.1) annotation file. Homologous mapping to the zebrafish genome (Danio rerio, GRCz11) was performed using BLASTn (v2.12.0) with an E-value threshold of 1 × 10^−5^, and KEGG pathway enrichment analysis was conducted using KOBAS (v3.0) with a hypergeometric test. *p*-values were corrected by the Benjamini–Hochberg method (corrected p<0.05) (These SNPs were selected for candidate gene annotation rather than representing independently detected genome-wide significant associations). Bar plots and network diagrams were generated using ggplot2(v3.4.4).

## 3. Result

### 3.1. Genetic Correlations Among Growth Traits

Table 1 presents the genetic correlation coefficients among the 11 growth traits of Japanese flounder (*Paralichthys olivaceus*). The standard errors of these estimates ranged from 0.016 to 0.318. The most precise estimates were observed for HL–PoL (SE = 0.016) and BW–BD (SE = 0.019), while the least precise were for SnL–ED (SE = 0.318) and ED–IW (SE = 0.288). Body weight (BW) exhibited high positive genetic correlations with most body size traits; the highest correlation was with body depth (BD, $r_{g}=0.992$), and very strong correlations were also observed with total length (TL, 0.951), trunk length (TUL, 0.945), and caudal peduncle depth (CPD, 0.993). Total length also showed high genetic correlations with body length (0.861), body depth (0.802), and trunk length (0.843). Among head measurements, head length (HL) and post-orbital head length (PoL) displayed an extremely strong genetic correlation (0.986), and HL was also highly correlated with eye diameter (ED, 0.971). Notably, eye diameter showed weak correlations with body depth (0.115) and caudal peduncle depth (0.054) and only a moderate correlation with body weight (0.397). Caudal peduncle depth (CPD) exhibited a remarkably high genetic correlation with interorbital width (IW, 0.994) and almost perfect correlation with body weight (0.993).

**Figure 1:**
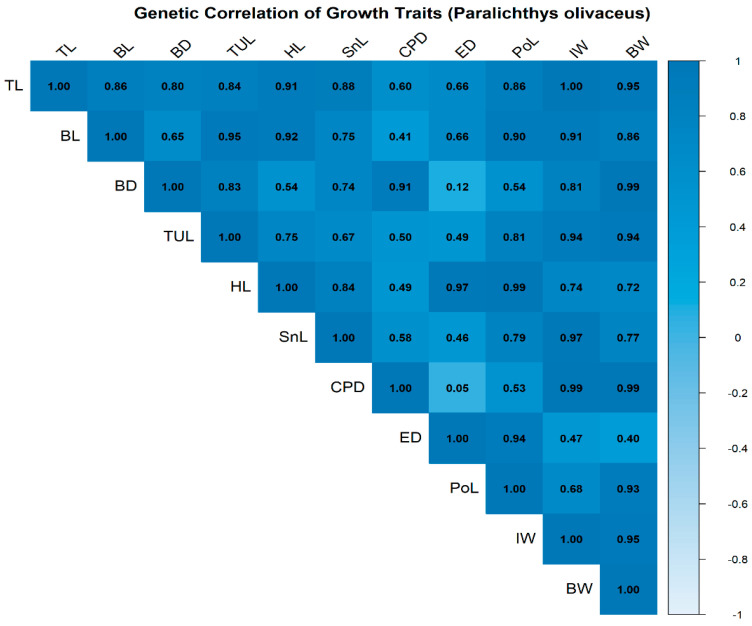
Genetic Correlation Matrix of Growth Traits in Japanese flounder (*Paralichthys olivaceus*).

Figure 1 visualizes the genetic correlation matrix. Deep blue blocks indicate strong positive correlations ($r_{g}>0.8$) among core body size traits—total length, body length, body depth, trunk length and caudal peduncle depth—and also among head traits (head length, post-orbital head length and interorbital width).

### 3.2. Descriptive Statistics and Heritability Estimates

Table 2 presents the descriptive statistics, heritability estimates and their standard errors for the 11 growth traits of Japanese flounder (*Paralichthys olivaceus*). The degrees of phenotypic variation for the different traits were not the same, and the coefficients of variation (CVs) ranged from 11.37% for total length (TL) to 33.99% for body weight (BW). Body weight, postorbital head length (PoL) and eye diameter (ED) had relatively large CVs (33.99%, 20.53% and 16.73%, respectively). The traits of body size (TL, BL, BD, TUL) had relatively small CVs (11.37–12.75%), indicating relatively uniform distribution of these measurements across individuals.

Heritability estimates showed that head length (HL) had the highest heritability ($h^{2}=0.487\pm 0.123$), which is considered moderate. Post-orbital head length (PoL), eye diameter (ED), body depth (BD) and caudal peduncle depth (CPD) followed, with heritabilities ranging from 0.291 to 0.328, also at moderate levels. Body weight (BW) and body length (BL) had heritabilities of 0.237 and 0.243, respectively, consistent with previous reports in olive flounder (0.12–0.48) [13,14,15]. Total length (TL), snout length (SnL), trunk length (TUL) and interorbital width (IW) exhibited lower heritabilities (0.172–0.209), with interorbital width having the lowest value (0.172 ± 0.083). Overall, head-related traits (HL, PoL, ED) showed higher heritability than body size traits. By contrast, the heritability of economically important traits such as body weight and body length is moderate to low.

**Figure 2:**
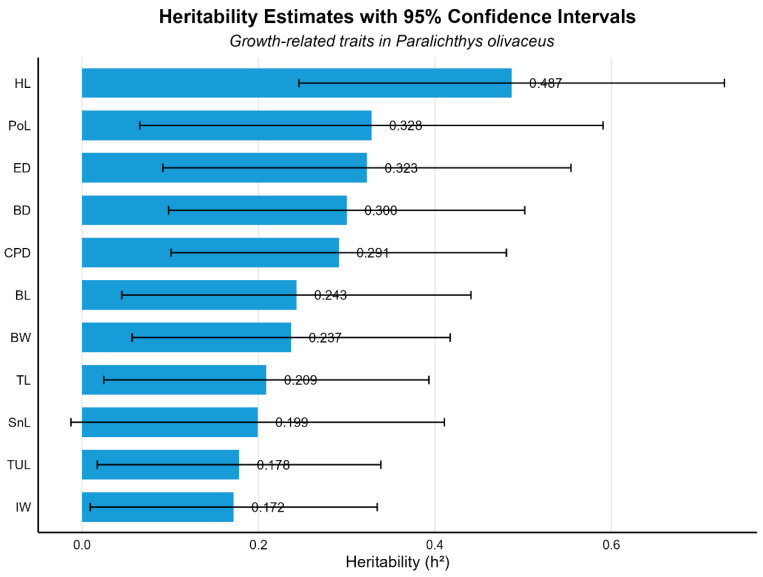
Ranking of heritability for growth traits in Japanese flounder (*Paralichthys olivaceus*).

Figure 2 presents a bar plot that visualizes the heritability estimates (with 95% confidence intervals) for the 11 growth traits, with traits arranged from highest to lowest heritability from top to bottom. Head length (HL) exhibits markedly higher heritability than the other traits, represented by the longest bar. Post-orbital head length (PoL), eye diameter (ED) and body depth (BD) follow closely, with bars of similar lengths forming the second tier. Total length (TL), snout length (SnL), trunk length (TUL) and interorbital width (IW) have shorter bars, indicating lower heritability, and interorbital width (IW) shows the shortest bar. The error bars represent the 95% confidence intervals and reflect the precision of the heritability estimates; the confidence interval for head length (HL) is relatively narrow.

### 3.3. Population Genetic Diversity

Principal component analysis based on 6.67 million high-quality SNPs (Table 3) showed that the first three principal components (PC1–PC3) explained 19.195%, 17.046% and 14.136% of the total variation, respectively, with a cumulative contribution of 50.376%. The first five principal components (PC1–PC5) cumulatively explained 71.439% of the variation, while the first ten principal components together accounted for 100% of the total variation. Regarding eigenvalues, PC1 had the highest eigenvalue (23.083), followed by a gradual decline, with PC10 decreasing to 4.956, a trend reflecting the successively diminishing amount of information carried by each principal component.

**Figure 3:**
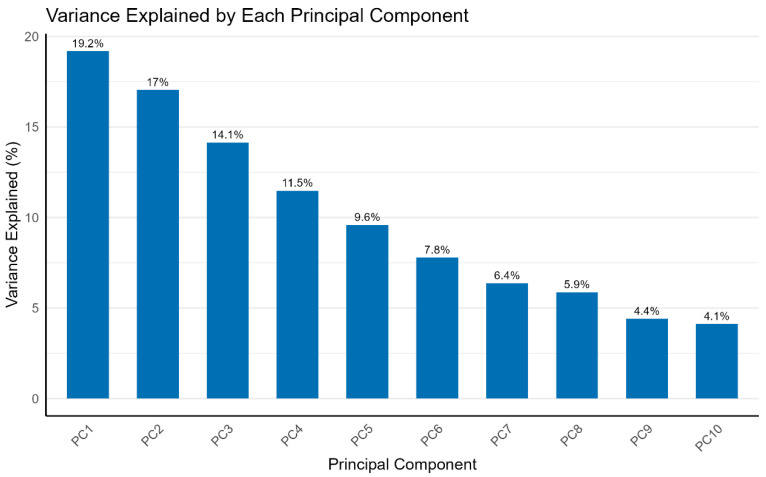
PCA Scree plot.

Figure 3 presents the scree plot of the eigenvalues for all principal components. The eigenvalues showed a sharp decrease from PC1 to PC3, followed by a continued but more gradual decline through PC4 and PC5, after which the decline became relatively flat. The first three principal components cumulatively explained 50.38% of the total variation. Based on this distribution, PC1–PC3 were retained as covariates in subsequent analyses to balance dimensionality reduction with information preservation.

The genetic diversity indices are summarized in Table 4. Based on the above results, it can be observed that the cultivated population has relatively low genetic diversity, and the observed heterozygosity is slightly lower than expected due to a certain degree of inbreeding or selection. The mean of the inbreeding coefficient was 0.049, but there was considerable individual variation; some individuals had an F value of 1.000, and thus the degree of inbreeding in the population was uneven.

### 3.4. Single-Trait GWAS Results

**Figure 4:**
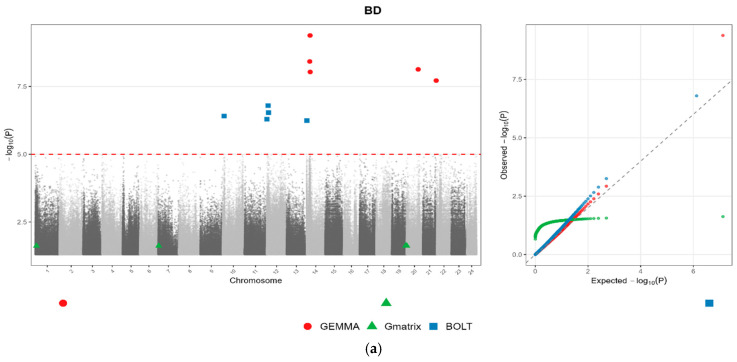

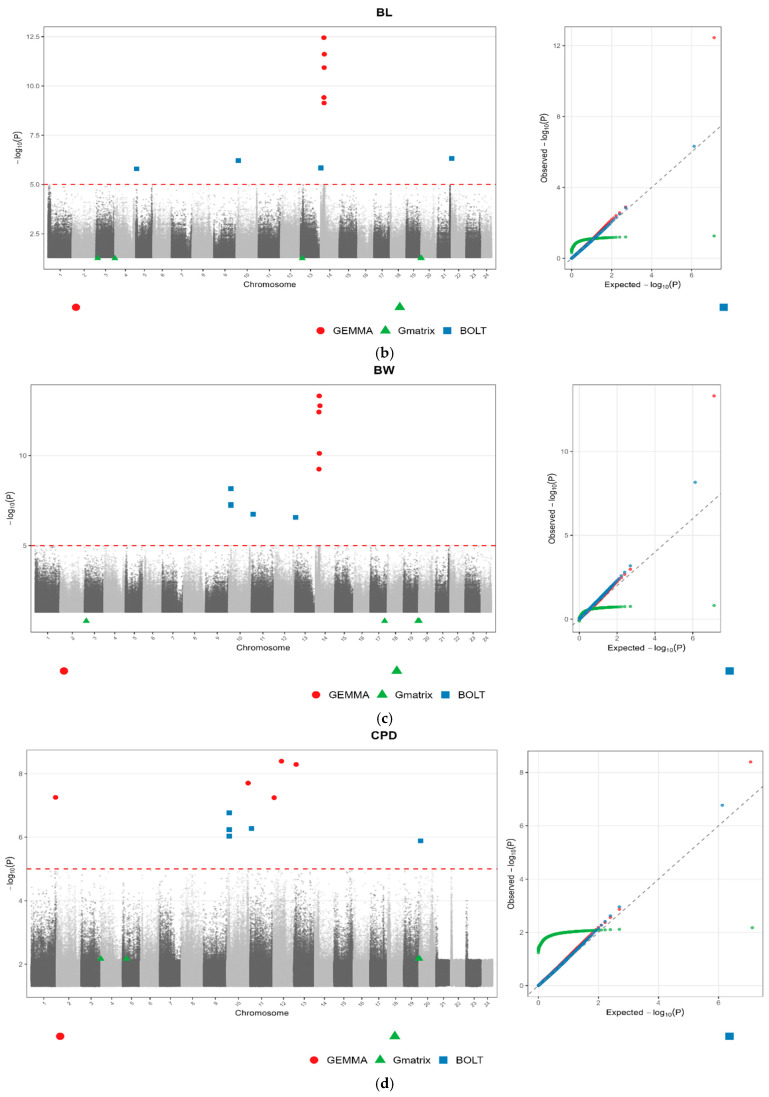

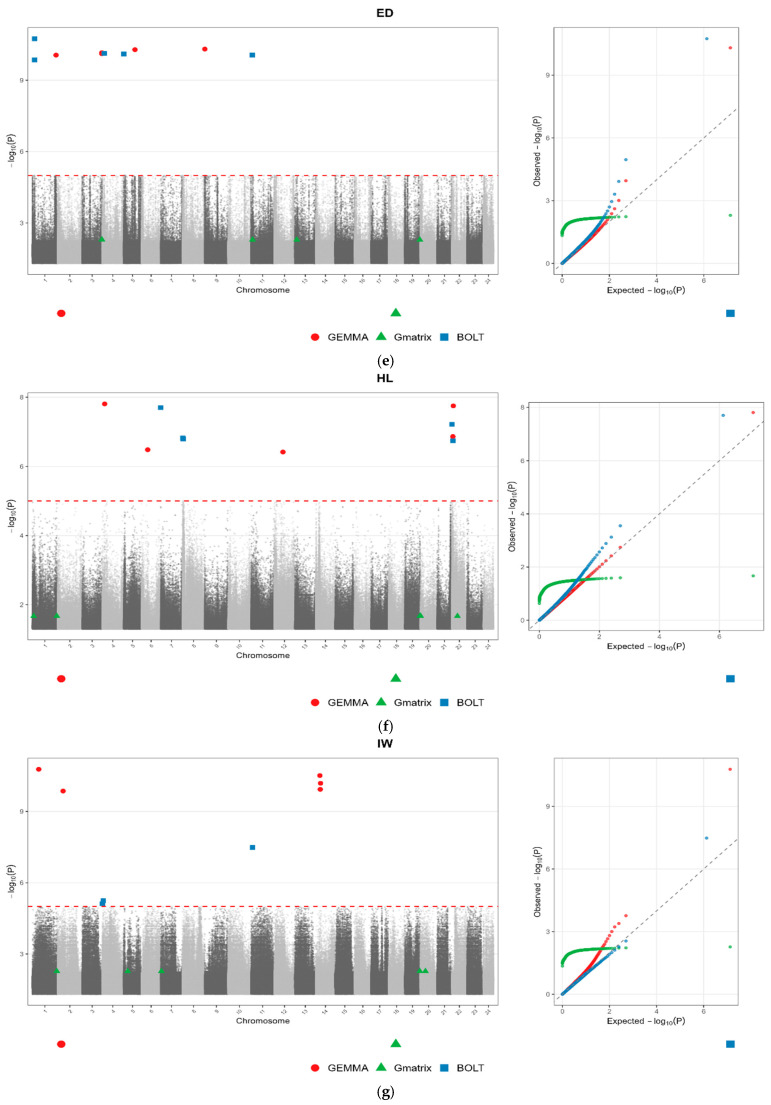

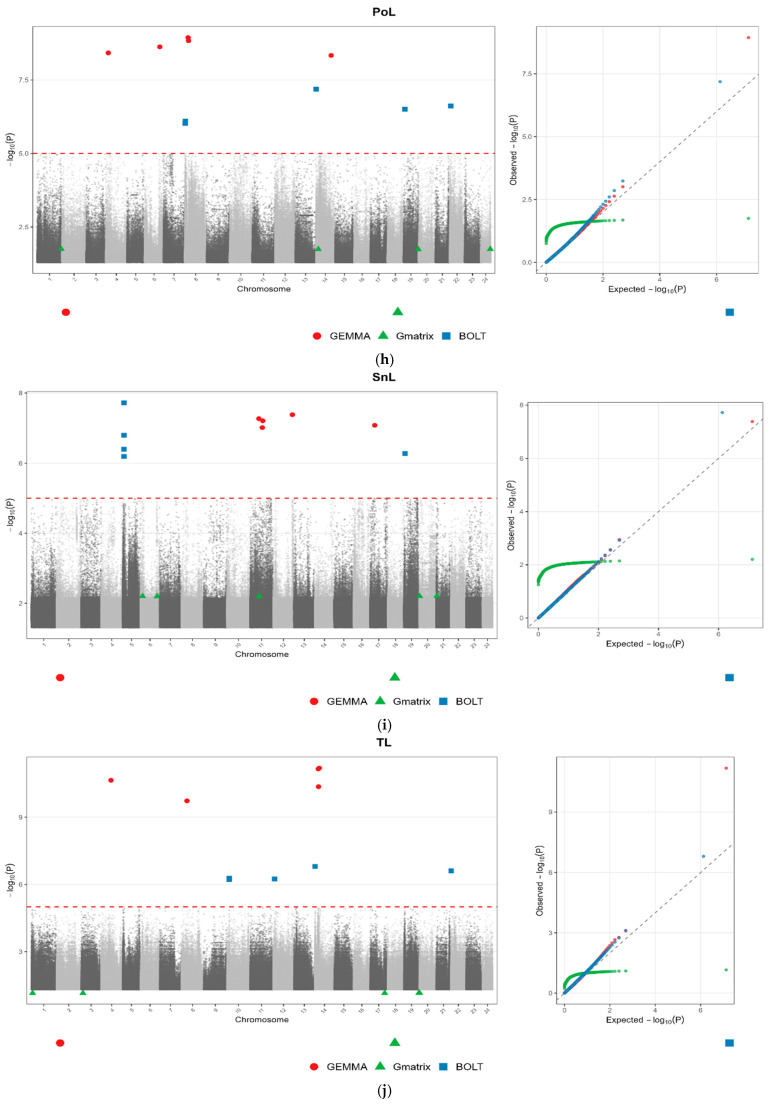

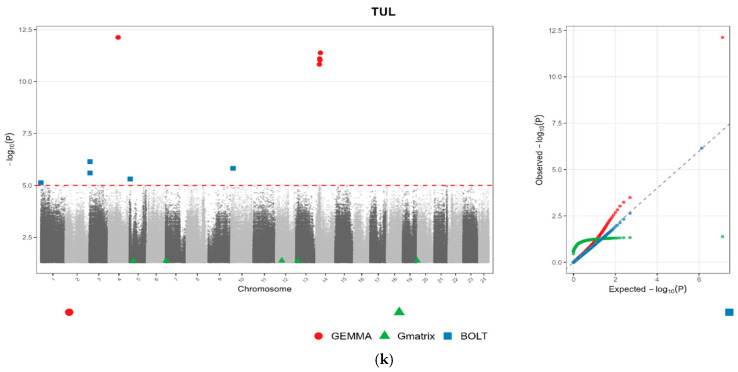
Genome-wide association study (GWAS) results for the 11 growth traits of Japanese flounder (*Paralichthys olivaceus*). Manhattan plots (**left**) and Q-Q plots (**right**) are shown for (**a**) body weight (BW), (**b**) total length (TL), (**c**) body length (BL), (**d**) body depth (BD), (**e**) trunk length (TUL), (**f**) head length (HL), (**g**) snout length (SnL), (**h**) caudal peduncle depth (CPD), (**i**) eye diameter (ED), (**j**) post-orbital head length (PoL), and (**k**) interorbital width (IW). The horizontal dashed line indicates the genome-wide suggestive significance threshold ($p=1\times 10^{-5}$). The red horizontal line indicates the genome-wide significant threshold ($p=5\times 10^{-8}$).

Single-trait GWAS results for all 11 growth traits are visualized in Figure 4. The genomic inflation factors (λ) for all traits ranged from 0.947 to 1.079, indicating that population stratification was well-controlled. A total of 55 suggestive associated loci (top five SNPs per trait, *p* < 1 × 10^−5^) were detected by single-trait GWAS, distributed on chromosomes 1, 2, 3, 4, 5, 6, 7, 8, 9, 10, 11, 12, 13, 14, 17, 20, 21 and 22 (Table 5). Among these, 31 loci reached the genome-wide significant threshold after Bonferroni correction (*p* < 7.5 × 10^−9^). Core growth traits (TL, BL, BD, BW) showed very strong association signals; for example, the most significant SNP for TL (14:6852741) had a *p*-value of 6.50 × 10^−12^. Among the software packages, GEMMA detected the largest number of loci (45), followed by BOLT-LMM (5) and GmatS (5), reflecting differences in algorithm sensitivity.

Several highly correlated traits shared the same genomic region. Several SNPs on chromosome 14 (14:6124961, 14:5853031, 14:6852741, 14:6275035, 14:5886236) have frequently been associated with more than five traits (TL, BL, BD, TUL, BW). Its closest genes are *LOC109631895* (uncharacterised), *LOC109634870* (GABA receptor π subunit), *dntt* (terminal deoxynucleotidyl transferase), *LOC138413661* (uncharacterised) and *LOC109629638* (adhesion G protein-coupled receptor A3).

Furthermore, the most significant SNP for head length (HL) was located within the *fras1* gene on chromosome 4 ($p=1.56\times 10^{-8}$). The significant SNP for interorbital width (IW) was located within the nkd1 gene on chromosome 1 ($p=1.67\times 10^{-11}$). Some SNPs were located inside genes (e.g., *stpg2*, *tfam*, *opn3*, *tial1*), while others were in intergenic regions (e.g., 14:5853031 is approximately 15 kb from *LOC109634870*).

### 3.5. Multi-Trait GWAS Results

To further uncover genetic loci that simultaneously affect multiple growth traits, multi-trait GWAS was performed on four trait combinations using three methods: GEMMA_mv, MultiTrait_mv and GmatS_mv, with a significance threshold of (*p* < 1 × 10^−5^). A total of 20 significant associated loci (top 5 SNPs per combination) were detected across the four combinations, distributed on chromosomes 1, 4, 5, 6, 7, 12, 14 and 22 (Table 6).

**Figure 5:**
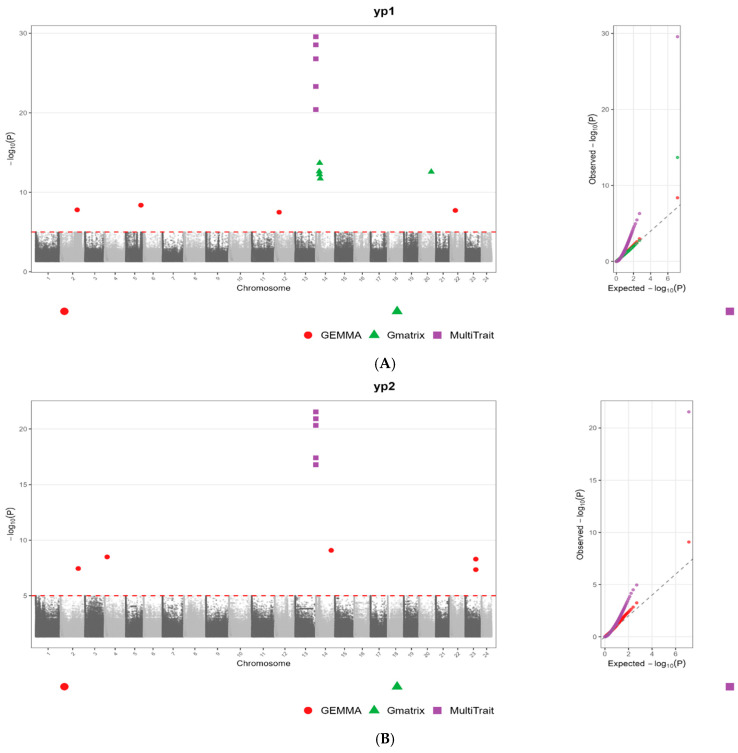

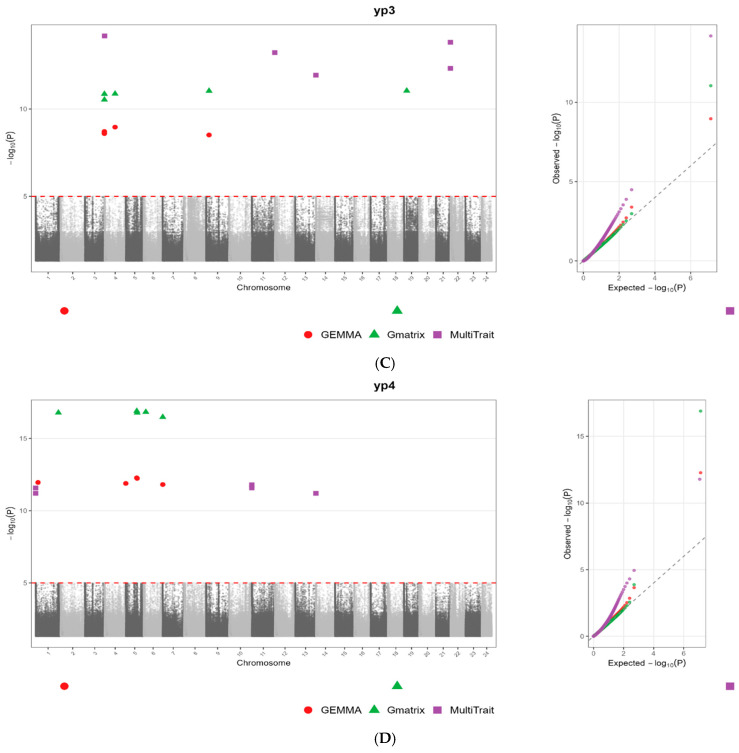
Multi-trait GWAS results for four trait combinations in Japanese flounder (*Paralichthys olivaceus*). Manhattan plots (**left**) and Q-Q plots (**right**) are shown for (**A**) combination 1 (TL + BL + BW), (**B**) combination 2 (BL + BD), (**C**) combination 3 (HL + SnL + PoL), and (**D**) combination 4 (ED + IW + CPD). The horizontal dashed line indicates the genome-wide suggestive significance threshold (*p* = 1 × 10^-5^). The red horizontal line indicates the genome-wide significant threshold (*p* = 5 × 10^−8^).

Multi-trait joint GWAS results of four morphological trait sets are displayed in Figure 5. The two main sites of combination 1 (TL + BL + BW) and combination 2 (BL + BD) are on chromosome 14, and these were in good agreement with the single-trait GWAS results. The same loci (14:6124961, 14:5853031, 14:6852741, 14:6275035, and 14:5886236) were found multiple times in many combinations, and the corresponding genes were the same as those mentioned above.

Combination 3 (HL + SnL + PoL) and Combination 4 (ED + IW + CPD) were new loci that did not reach the significance level in the single-trait analysis. For example, in Combination 3, the locus on chromosome 4 identified by MultiTrait_mv is in the *fras1* gene. This gene had already been identified in the single-trait GWAS for head length, but it was even more pronounced in this combination. The locus on chromosome 22 is near LOC109637704 (histone H2A.Z). In addition, the locus on chromosome 12 is close to LOC138412244 (histone H1-like), and the locus on chromosome 14 is in the *kcnq5a* (potassium voltage-gated channel) gene. In combination 4, the loci on chromosomes 5, 6, 1 and 7 that were detected by GmatS_mv are associated with genes such as *LOC138407752* (sodium channel protein α subunit) and *lamb4* (laminin β4).

Among the 20 loci, 12 did not reach the significance level of in the single-trait analysis (e.g., the *p*-value of *fras1* in combination 3 was much lower than the single-trait threshold). MultiTrait_mv had the highest number of loci (15) and the smallest *p*-values, followed by GEMMA_mv (5), and GmatS_mv found 5 loci (some of which had a coordinate of 0; this may be due to a different version of the reference genome). To complement single-trait GWAS and uncover pleiotropic loci, we performed multi-trait joint analysis, which revealed additional results on the genetic structure of growth traits in Japanese flounder.

### 3.6. KEGG Pathway Enrichment Analysis

**Figure 6:**
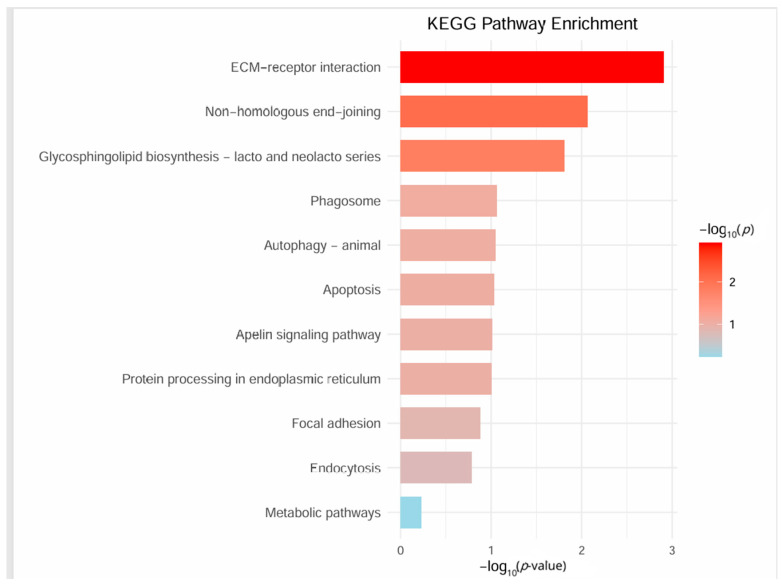
Bar plot of KEGG pathway enrichment. Note: The horizontal axis is the enrichment ratio (number of input genes/total genes in the pathway × 100%), and the vertical axis lists KEGG pathway names in ascending order of *p*-value (most significant at the top). The colour gradient is −log_10_(*p*), and a darker colour is more significant. On the right side of each bar, the number of input genes/total genes and the *p*-value are marked.

KEGG enrichment analysis showed that the candidate genes were enriched in 11 pathways, of which two reached statistical significance (corrected p<0.05, Table 7). The most significant pathway was “ECM–receptor interaction” (corrected p=0.0135), containing two candidate genes (*fras1* and *lamb4*), with an enrichment ratio of 2.25% (Figure 6). The other significant pathway was “Non-homologous end-joining” (corrected p=0.0470), containing one candidate gene (*dntt*), with an enrichment ratio of 7.14%.

**Figure 7:**
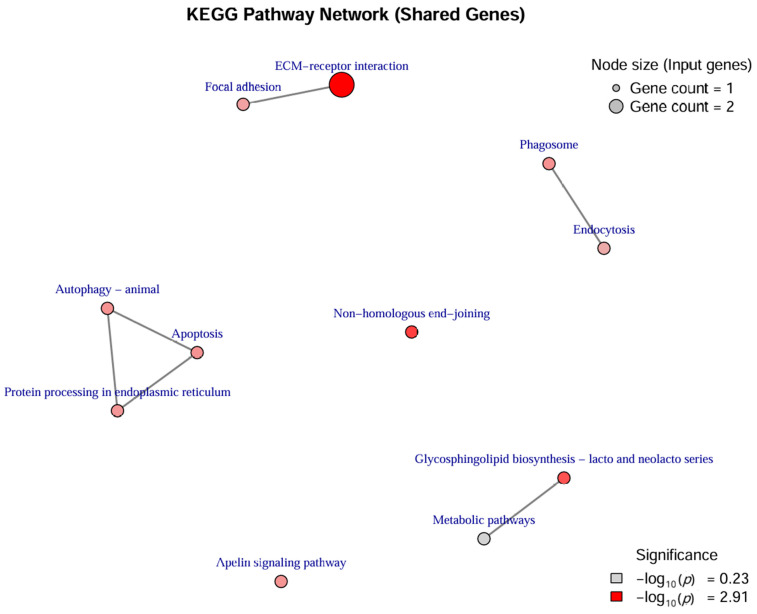
KEGG pathway network of shared genes. Note: Nodes are KEGG pathways; the size of the node is the number of input genes; the colour of the node shows significance (−log_10_(*p*)), and darker red is more significant. The edges show that at least one candidate gene is shared among pathways, and the thickness of the edge is the number of shared genes.

Figure 7 is the pathway network diagram of shared genes. Based on the above results, it can be concluded that *fras1* and *lamb4* work together in the “ECM–receptor interaction” pathway and are both components of the extracellular matrix; *dntt* is independently involved in the “Non-homologous end-joining” pathway. There are no complicated connections of multiple paths sharing the same gene in the network diagram; it is possible that the number of candidate genes in this study was relatively small. However, the enriched pathways have been strengthened; thus, it can be concluded that extracellular matrix homeostasis and DNA damage repair may be involved in the growth of Japanese flounder (*Paralichthys olivaceus*), and further functional validation and the development of markers for marker-assisted selection should be carried out in the next step.

## 4. Discussion

Genetic architecture analysis of the 11 growth traits in Japanese flounder (*Paralichthys olivaceus*) has been carried out in this paper by means of single-trait and multi-trait GWAS to identify a group of significant candidate genes and their corresponding biological pathways that can be used as potential targets for marker-assisted selection.

### 4.1. Genetic Parameters of Growth Traits

In the present study, head length (HL) had the highest heritability ($h^{2}=0.487$), followed by post-orbital head length (PoL), eye diameter (ED), body depth (BD) and caudal peduncle depth (CPD) (0.291–0.328), all of which are at moderate heritability levels, consistent with previous reports of relatively high heritability for head morphology traits in olive flounder [13,14,15]. Body weight (BW) and body length (BL) had heritabilities of 0.237 and 0.243, respectively, which fall within the previously estimated range (0.12–0.48) for olive flounder [13,14,15], indicating that these economic traits still possess certain selection potential. Total length (TL), snout length (SnL), trunk length (TUL) and interorbital width (IW) exhibited lower heritabilities (0.172–0.209), suggesting that progress based solely on phenotypic selection may be slow and that marker-assisted selection should be employed. Head-related traits (HL, PoL, ED) showed higher heritability than body size traits, a phenomenon also observed in rainbow trout [22], implying that head morphology is under stronger additive genetic control, whereas body size traits are more easily influenced by environmental factors.

Genetic correlation analysis revealed that body weight was highly positively correlated with most body size traits ($r_{g}>0.9$), which is consistent with results from Atlantic salmon [23] and common carp [5], supporting multi-trait synergistic improvement. Particularly noteworthy, caudal peduncle depth (CPD) was almost perfectly correlated with body weight ($r_{g}=0.993$) and with interorbital width (IW, $r_{g}=0.994$), suggesting that these traits may be controlled by nearly identical genetic loci and can serve as indirect selection indicators for each other in breeding. Eye diameter (ED) showed very weak correlations with body depth (BD) and caudal peduncle depth (CPD) ($r_{g}<0.12$) and only a moderate correlation with body weight (0.397), indicating relatively independent genetic regulation of eye diameter. This phenomenon is reported here for the first time in fish and may be related to the unique flattened body shape and specialised eye development of Japanese flounder (*Paralichthys olivaceus*).

The first three principal components explained about 50.38% of the variation in the population genetic analysis. The mean observed heterozygosity (Ho = 0.206) was slightly lower than the expected heterozygosity (He = 0.216), and although the mean inbreeding coefficient (F = 0.049) was small, there was a large range in interindividual variation of F (−0.695 to 1.000). Overall, the cultured population exhibits moderate genetic diversity and is not severely affected by inbreeding depression, although some individuals show high inbreeding coefficients. Long-term monitoring of genetic diversity is advised.

### 4.2. Single-Trait GWAS Candidate Genes

Single-trait GWAS found 55 significant loci, and among them, some SNPs in the region of chromosome 14 (14:6124961, 14:5853031, 14:6852741, 14:6275035 and 14:5886236) were frequently associated with more than five traits (TL, BL, BD, TUL, BW). Interestingly, a QTL hotspot on chromosome 14 has not been reported in the previous GWAS on Japanese flounder by Omeka et al. (2022) [20]. This discrepancy may be due to differences in population structure, marker density, or sample size, and warrants further investigation. Thus, this region represents a pleiotropic QTL hotspot and affects many growth traits. It should be noted that GWAS identifies markers in linkage disequilibrium with causal variants, not the causal genes themselves. Therefore, the candidate genes discussed here are putative targets that require further fine-mapping and functional validation. The neighbouring genes are *LOC109634870* (GABA receptor π subunit), *dntt* (terminal deoxynucleotidyl transferase) and *LOC109629638* (adhesion G protein-coupled receptor A3). The GABA receptor is involved in nerve signalling and may affect growth by regulating feeding behaviour and energy metabolism [29]; *dntt* is a necessary DNA repair enzyme that has been associated with cell division and is linked to changes in body weight and length; the adhesion G protein-coupled receptor A3 is involved in cell adhesion and signal transmission, and it is also related to the growth of muscle and bone. In addition, the most obvious SNP for head length (HL) is in the *fras1* gene. The association of *fras1* with head length is consistent with its known role in craniofacial development in mammals [29], but its function in fish growth has not been previously reported. it is a protein that is part of the extracellular matrix (Fraser complex subunit 1) and involved in the development of basement membranes and morphogenesis; thus, mutations in this gene are associated with craniofacial abnormalities in humans and mice [29]. The considerable SNP in interorbital width (IW) is situated in the nkd1 gene; it is an inhibitor of the WNT signalling pathway that is involved in all stages of embryonic development, organ growth and adult tissue maintenance [30]. These findings expand the list of candidate genes potentially regulating growth in Japanese flounder.

### 4.3. New Loci from Multitrait GWAS

Multi-trait GWAS identified 20 loci, of which 12 did not reach significance in single-trait analyses, highlighting the unique advantage of multi-trait approaches in uncovering pleiotropic loci. In combination 3 (HL + SnL + PoL), the significance of the *fras1* locus was further increased ($p=6.50\times 10^{-15}$), confirming the central role of this gene in head morphology development. In addition, loci near LOC109637704 (histone H2A.Z) on chromosome 22 and LOC138412244 (histone H1-like) on chromosome 12 were detected, suggesting that epigenetic regulation (histone variant modifications) may be involved in head trait formation [31]. In combination 4 (ED + IW + CPD), GmatS_mv detected the *lamb4* (laminin β4) locus; laminins are important extracellular matrix components involved in cell adhesion, migration and signal transduction, and are closely related to muscle and bone development [32]. These multi-trait-specific loci provide new clues for dissecting the complex genetic network underlying growth traits in Japanese flounder (*Paralichthys olivaceus*). The classical growth-related genes *mstn* and *ghr* were not detected as significant in this study, possibly due to the relatively small sample size (282 individuals) limiting power to detect small-effect variants.

### 4.4. Pathway Enrichment Analysis

Based on the enrichment analysis of KEGG, it was found that the candidate genes are enriched in the pathways of “ECM–receptor interaction” and “Nonhomologous end-joining”. The ECM–receptor interaction pathway is needed for the adhesion, migration and signal transmission of cells on the extracellular matrix, so it is related to the formation and modification of bone, muscle, etc., connective tissues [33]. The two genes in this pathway are *fras1* [34] and *lamb4*; both are components of the extracellular matrix, and their coexpression may affect the growth of the body trunk and the shape of the head in Japanese flounder (*Paralichthys olivaceus*). Non-homologous end-joining is the primary repair pathway for double-strand breaks in DNA, and it is related to cell division, genome stability and the rate of individual cell growth [34]. Therefore, *dntt* may also promote growth through DNA repair regulation. The other nine paths (phagosome, autophagy, apoptosis, etc.) were not significant, but they may still affect growth and need to be further studied. The pathway network did not have many links through shared genes; it is probably because there are not enough candidate genes in this study.

### 4.5. Implications for Breeding and Future Directions

The genetic parameters and associated loci identified in this study have potential applications in Japanese flounder breeding programs. The high genetic correlations between body weight and other body size traits (e.g., BD, CPD) suggest that indirect selection for a readily measurable trait such as body depth could effectively improve body weight without the need for direct measurement of weight in every generation. The suggestive QTLs on chromosome 14, particularly those near *dntt* and GABA receptor genes, provide potential targets for developing SNP markers that could be incorporated into genomic selection pipelines. However, before these markers can be deployed in commercial breeding, they must be validated in larger, independent populations and, ideally, across different genetic backgrounds.

Future work should focus on: (1) fine-mapping the chromosome 14 region using higher-density markers to narrow down the candidate interval; (2) conducting functional experiments (e.g., CRISPR/Cas9 knockout or overexpression) to confirm the roles of candidate genes such as *fras1* and *lamb4* in growth regulation; and (3) evaluating the predictive accuracy of these markers in independent populations to assess their practical utility in marker-assisted selection.

## 5. Limitations

The deficiencies of this study are as follows: (1) The sample size (N = 282) is relatively small; thus, the statistical power may be low, and small-effect genes, especially for traits with low heritability (such as interorbital width and trunk length), may not be included in the association results. (2) The functional annotation is based on homology mapping with zebrafish, and due to the lack of complete annotation for the Japanese flounder (*Paralichthys olivaceus*) genome, the actual function of some genes may differ from the prediction and needs to be verified experimentally (e.g., in situ hybridization, gene knockout). (3) KEGG enrichment analysis only considers well-known pathways and may fail to identify fish-specific growth regulatory pathways. (4) No functional validation experiments have been carried out to determine whether the candidate gene is indeed associated with the growth trait. (5) All samples are from the same farm, and it has not been verified how generalizable the results are to other genetic backgrounds.

## 6. Conclusions

This study performed genome-wide association analyses for 11 growth traits in 282 Japanese flounder (*Paralichthys olivaceus*) individuals and drew the following main conclusions: (1) Head length had the highest heritability (0.487), body weight and body length had moderate heritability (0.237 and 0.243), body size traits had moderate-to-low heritability (0.172–0.300), and head traits generally showed higher heritability than body size traits. Body weight was highly positively correlated with most body size traits ($r_{g}>0.9$), supporting multi-trait synergistic improvement. (2) Single-trait GWAS identified 55 significant loci and revealed a pleiotropic QTL hotspot on chromosome 14, with nearby genes including the GABA receptor π subunit and *dntt*. *fras1* was significantly associated with head length, and nkd1 with interorbital width. (3) Multi-trait GWAS discovered 12 novel loci that were not significant in single-trait analyses, involving core genes such as *fras1*, *lamb4* and histone family members, effectively complementing the single-trait results. (4) KEGG enrichment analysis showed that candidate genes were significantly enriched in the “ECM–receptor interaction” and “Non-homologous end-joining” pathways, highlighting the potential important roles of extracellular matrix dynamics and DNA damage repair in Japanese flounder (*Paralichthys olivaceus*) growth. (5) The core candidate genes identified (*fras1*, *dntt*, *lamb4*, *kcnq5a*, etc.) can serve as potential targets for marker-assisted selection in Japanese flounder (*Paralichthys olivaceus*), providing an important foundation for multi-trait synergistic improvement.

## Figures and Tables

**Table 1:** Genetic correlations among 11 growth traits in Japanese flounder.

	TL	BL	BD	TUL	HL	SnL	CPD	ED	PoL	IW	BW
TL	1	0.861	0.802	0.843	0.908	0.882	0.604	0.664	0.864	0.998	0.951
SE		(0.073)	(0.109)	(0.094)	(0.072)	(0.122)	(0.193)	(0.220)	(0.143)	(0.057)	(0.036)
BL		1	0.652	0.947	0.924	0.748	0.405	0.663	0.904	0.909	0.857
SE			(0.152)	(0.040)	(0.054)	(0.179)	(0.213)	(0.217)	(0.106)	(0.115)	(0.082)
BD			1	0.833	0.543	0.742	0.906	0.115	0.544	0.808	0.992
SE				(0.113)	(0.183)	(0.179)	(0.085)	(0.278)	(0.241)	(0.163)	(0.019)
TUL				1	0.753	0.673	0.504	0.487	0.815	0.937	0.945
SE					(0.166)	(0.226)	(0.241)	(0.284)	(0.212)	(0.119)	(0.051)
HL					1	0.838	0.490	0.971	0.986	0.743	0.723
SE						(0.133)	(0.196)	(0.071)	(0.016)	(0.177)	(0.142)
SnL						1	0.585	0.462	0.786	0.968	0.773
SE							(0.264)	(0.318)	(0.189)	(0.146)	(0.163)
CPD							1	0.054	0.526	0.994	0.993
SE								(0.276)	(0.237)	(0.081)	(0.033)
ED								1	0.935	0.469	0.397
SE									(0.126)	(0.288)	(0.270)
PoL									1	0.678	0.929
SE										(0.247)	(0.207)
IW										1	0.954
SE											(0.076)
BW											1

Note: Values above the diagonal are genetic correlation coefficients (r_g); values below the diagonal (in parentheses) are standard errors (SE). All estimates were obtained from bivariate GREML analyses using GCTA (v1.94.1). TL = total length, BL = body length, BD = body depth, TUL = trunk length, HL = head length, SnL = snout length, CPD = caudal peduncle depth, ED = eye diameter, PoL = postorbital head length, IW = interorbital width, BW = body weight.

**Table 2:** Descriptive Statistics and Heritability Estimates of Growth Traits in Japanese flounder (*Paralichthys olivaceus*).

Trait	N_Individuals	Mean	Sd	CV (%)	h^2^	Se
BW	282	6.355	2.160	33.99	0.237	0.092
TL	282	8.399	0.955	11.37	0.209	0.094
BL	282	6.522	0.754	11.56	0.243	0.101
BD	282	2.773	0.334	12.05	0.300	0.103
TUL	282	4.421	0.564	12.75	0.178	0.082
HL	282	2.101	0.291	13.87	0.487	0.123
SnL	282	0.523	0.084	16.08	0.199	0.108
CPD	282	0.679	0.096	14.17	0.291	0.097
ED	282	0.409	0.068	16.73	0.323	0.118
PoL	282	1.169	0.240	20.53	0.328	0.134
IW	282	0.569	0.073	12.84	0.172	0.083

Note: The unit of size for body traits is cm; BW is in grams.

**Table 3:** PCA Results for 282 Samples.

Principal Component	Eigenvalue	Variance Explained (%)	Cumulative (%)
PC1	23.083	19.195	19.195
PC2	20.498	17.046	36.240
PC3	16.999	14.136	50.376
PC4	13.801	11.481	61.858
PC5	11.522	9.581	71.439
PC6	9.369	7.790	79.229
PC7	7.661	6.371	85.599
PC8	7.061	5.871	91.471
PC9	5.300	4.408	95.878
PC10	4.956	4.121	100

**Table 4:** Genetic Diversity Indices of All Samples.

Parameter	Value
Number of individuals	282
Mean observed heterozygosity (Ho)	0.206
Mean expected heterozygosity (He)	0.216
Mean inbreeding coefficient (F)	0.049
Ho range	0–0.367
He range	0.216–0.216
F range	−0.695–1

**Table 5:** Top 5 significant SNPs for each growth trait in single-trait GWAS.

Trait	Software	CHR	Pos	*p*_Value	Nearest_Gene	Gene Description	Distance to Gene
TL	GEMMA	14	6,852,741	6.50 × 10^−12^	*LOC138413661*	uncharacterized *LOC138413661*	0
TL	GEMMA	14	5,853,031	7.01 × 10^−12^	*LOC109634870*	gamma-aminobutyric acid receptor subunit pi	14,932
TL	GEMMA	4	11,862,924	2.26 × 10^−11^	*stpg2*	sperm-tail PG-rich repeat containing 2	8891
TL	GEMMA	14	6,124,961	4.37 × 10^−11^	*LOC109631895*	uncharacterized *LOC109631895*	537
TL	GEMMA	8	5,802,526	1.88 × 10^−10^	*LOC138411296*	uncharacterized *LOC138411296*	42,641
BL	GEMMA	14	5,853,031	3.55 × 10^−13^	*LOC109634870*	gamma-aminobutyric acid receptor subunit pi	14,932
BL	GEMMA	14	6,275,035	2.44 × 10^−12^	*dntt*	deoxynucleotidyltransferase, terminal	0
BL	GEMMA	14	6,124,961	1.17 × 10^−11^	*LOC109631895*	uncharacterized *LOC109631895*	537
BL	GEMMA	14	5,886,236	3.84 × 10^−10^	*LOC109629638*	adhesion G protein-coupled receptor A3	0
BL	GEMMA	14	6,069,741	7.33 × 10^−10^	*LOC109630372*	kinase non-catalytic C-lobe domain-containing protein 1	0
BD	GEMMA	14	6,124,961	4.15 × 10^−10^	*LOC109631895*	uncharacterized *LOC109631895*	537
BD	GEMMA	14	5,853,031	3.79 × 10^−9^	*LOC109634870*	gamma-aminobutyric acid receptor subunit pi	14,932
BD	GEMMA	20	14,240,253	7.39 × 10^−9^	*LOC109631500*	glutathione S-transferase A	110
BD	GEMMA	14	6,275,035	9.20 × 10^−9^	*dntt*	deoxynucleotidyltransferase, terminal	0
BD	GEMMA	21	18,090,729	1.92 × 10^−8^	*tfam*	transcription factor A, mitochondrial	1181
TUL	GEMMA	4	11,862,924	7.49 × 10^−13^	*stpg2*	sperm-tail PG-rich repeat containing 2	8891
TUL	GEMMA	14	6,852,741	4.15 × 10^−12^	*LOC138413661*	uncharacterized *LOC138413661*	0
TUL	GEMMA	14	6,124,961	7.92 × 10^−12^	*LOC109631895*	uncharacterized *LOC109631895*	537
TUL	GEMMA	14	6,275,035	9.15 × 10^−12^	*dntt*	deoxynucleotidyltransferase, terminal	0
TUL	GEMMA	14	5,853,031	1.48 × 10^−11^	*LOC109634870*	gamma-aminobutyric acid receptor subunit pi	14,932
HL	GEMMA	4	2,573,808	1.56 × 10^−8^	*fras1*	Fraser extracellular matrix complex subunit 1	0
HL	GEMMA	22	3,134,867	1.78 × 10^−8^	*LOC109637704*	histone H2A.Z	2166
HL	BOLT	7	126,334	2.00 × 10^−8^	*LOC138410832*	proteoglycan 4-like	0
HL	BOLT	22	3,401,602	6.10 × 10^−8^	*rap1gds1*	RAP1, GTP-GDP dissociation stimulator 1	0
HL	GEMMA	22	2,795,682	1.37 × 10^−7^	*LOC109637601*	membrane-spanning 4-domains subfamily A member 4A-like	83
SnL	BOLT	5	18,183,442	1.90 × 10^−8^	*ccdc97*	coiled-coil domain containing 97	671
SnL	GEMMA	12	24,250,341	4.16 × 10^−8^	*LOC138412244*	histone H1-like	2894
SnL	GEMMA	11	8,475,725	5.40 × 10^−8^	*st3gal4*	ST3 beta-galactoside alpha-2,3-sialyltransferase 4	0
SnL	GEMMA	11	13,354,973	6.22 × 10^−8^	*LOC109629945*	F-BAR and double SH3 domains protein 2	0
SnL	GEMMA	17	6,079,503	8.36 × 10^−8^	*mtrr*	5-methyltetrahydrofolate-homocysteine methyltransferase reductase	11,350
CPD	GEMMA	12	11,513,898	4.01 × 10^−9^	*LOC109636397*	MAPK/MAK/MRK overlapping kinase	0
CPD	GEMMA	13	2,776,129	5.09 × 10^−9^	*rab5ab*	RAB5A, member RAS oncogene family, b	0
CPD	GEMMA	10	22,133,994	1.97 × 10^−8^	*gpr39*	G protein-coupled receptor 39	0
CPD	GEMMA	1	30,874,108	5.56 × 10^−8^	*XM_069527633.1*	Paralichthys olivaceus golgin subfamily B member 1-like (LOC109646560), transcript variant X7, mRNA.	NA
CPD	GEMMA	12	3,006,472	5.69 × 10^−8^	*lgals3b*	lectin, galactoside binding soluble 3b	766
ED	BOLT	1	30,166,900	1.80 × 10^−11^	*XR_011243877.1*	Paralichthys olivaceus uncharacterized lncRNA,98.45%	NC_091093.1
ED	GEMMA	9	1,041,879	4.91 × 10^−11^	*LOC138411433*	uncharacterized LOC138411433	18,474
ED	GEMMA	5	16,338,322	5.20 × 10^−11^	*ighd*	immunoglobulin heavy constant delta	0
ED	GEMMA	3	27,555,922	6.93 × 10^−11^	*LOC138407274*	tissue factor-like	28,012
ED	BOLT	4	27,520,423	7.40 × 10^−11^	*LOC138407354*	uncharacterized LOC138407354	4097
PoL	GEMMA	8	3,725,182	1.15 × 10^−9^	*ern2*	endoplasmic reticulum to nucleus signaling 2	0
PoL	GEMMA	8	4,007,506	1.47 × 10^−9^	*LOC109644728*	dnaJ homolog subfamily A member 3, mitochondrial-like	855
PoL	GEMMA	6	24,336,663	2.36 × 10^−9^	*plxna2*	plexin A2	0
PoL	GEMMA	4	2,573,808	3.80 × 10^−9^	*fras1*	Fraser extracellular matrix complex subunit 1	0
PoL	GEMMA	14	20,950,472	4.63 × 10^−9^	*kcnq5a*	potassium voltage-gated channel, KQT-like subfamily, member 5a	0
IW	GEMMA	1	8,917,271	1.67 × 10^−11^	*XM_020080237.2*	Paralichthys olivaceus NKD inhibitor of WNT signaling pathway 1 (nkd1), mRNA	NA
IW	GEMMA	14	6,496,333	3.07 × 10^−11^	*opn3*	opsin 3	1651
IW	GEMMA	14	7,188,695	6.47 × 10^−11^	*tial1*	TIA1 cytotoxic granule-associated RNA binding protein-like 1	0
IW	GEMMA	14	6,852,741	1.17 × 10^−10^	*LOC138413661*	uncharacterized *LOC138413661*	0
IW	GEMMA	2	6,003,998	1.37 × 10^−10^	*XM_069532901.1*	Paralichthys olivaceus thyroid stimulating hormone subunit beta a (tshba), mRNA	NA
BW	GEMMA	14	6,124,961	4.98 × 10^−14^	*LOC109631895*	uncharacterized *LOC109631895*	537
BW	GEMMA	14	6,852,741	1.76 × 10^−13^	*LOC138413661*	uncharacterized *LOC138413661*	0
BW	GEMMA	14	5,853,031	3.91 × 10^−13^	*LOC109634870*	gamma-aminobutyric acid receptor subunit pi	14,932
BW	GEMMA	14	6,275,035	7.56 × 10^−11^	*dntt*	deoxynucleotidyltransferase, terminal	0
BW	GEMMA	14	5,886,236	5.69 × 10^−10^	*LOC109629638*	adhesion G protein-coupled receptor A3	0

**Table 6:** Top Five Significant SNPs for each Trait Combination in Multi-trait GWAS and their Gene Annotations.

Trait_Set	Method	CHR	Pos	*p*_Value	Nearest_Gene	Gene Function	Distance_to_Gene
1lei	MultiTrait_mv	14	6,124,961	2.68 × 10^−30^	*LOC109631895*	uncharacterized LOC109631895	537
1lei	MultiTrait_mv	14	5,853,031	2.89 × 10^−29^	*LOC109634870*	gamma-aminobutyric acid receptor subunit pi	14,932
1lei	MultiTrait_mv	14	6,852,741	1.61 × 10^−27^	*LOC138413661*	uncharacterized LOC138413661	0
1lei	MultiTrait_mv	14	6,275,035	4.91 × 10^−24^	*dntt*	deoxynucleotidyltransferase, terminal	0
1lei	MultiTrait_mv	14	5,886,236	3.99 × 10^−21^	*LOC109629638*	adhesion G protein-coupled receptor A3	0
2lei	MultiTrait_mv	14	5,853,031	2.87 × 10^−22^	*LOC109634870*	gamma-aminobutyric acid receptor subunit pi	14,932
2lei	MultiTrait_mv	14	6,275,035	1.19 × 10^−21^	*dntt*	deoxynucleotidyltransferase, terminal	0
2lei	MultiTrait_mv	14	6,124,961	4.80 × 10^−21^	*LOC109631895*	uncharacterized LOC109631895	537
2lei	MultiTrait_mv	14	6,852,741	3.76 × 10^−18^	*LOC138413661*	uncharacterized LOC138413661	0
2lei	MultiTrait_mv	14	5,886,236	1.56 × 10^−17^	*LOC109629638*	adhesion G protein-coupled receptor A3	0
3lei	MultiTrait_mv	4	2,573,808	6.50 × 10^−15^	*fras1*	Fraser extracellular matrix complex subunit 1	0
3lei	MultiTrait_mv	22	3,134,867	1.49 × 10^−14^	*LOC109637704*	histone H2A.Z	2166
3lei	MultiTrait_mv	12	24,250,341	5.88 × 10^−14^	*LOC138412244*	histone H1-like	2894
3lei	MultiTrait_mv	22	2,795,682	4.69 × 10^−13^	*LOC109637601*	membrane-spanning 4-domains subfamily A member 4A-like	83
3lei	MultiTrait_mv	14	20,950,472	1.15 × 10^−12^	*kcnq5a*	potassium voltage-gated channel, KQT-like subfamily, member 5a	0
4lei	GmatS_mv	5	0	1.27 × 10^−17^	*LOC138407752*	sodium channel protein type 4 subunit alpha B-like	3294
4lei	GmatS_mv	6	0	1.47 × 10^−17^	*LOC138410607*	uncharacterized LOC138410607	215,023
4lei	GmatS_mv	1	0	1.65 × 10^−17^	*LOC138411061*	uncharacterized LOC138411061	94,855
4lei	GmatS_mv	5	0	1.68 × 10^−17^	*LOC138407752*	sodium channel protein type 4 subunit alpha B-like	3294
4lei	GmatS_mv	7	0	3.30 × 10^−17^	*lamb4*	laminin, beta 4	15,537

**Table 7:** KEGG pathway enrichment analysis of candidate genes associated with growth traits in Japanese flounder.

Rank	Pathway	ID	Input_Genes	Total_Genes	Enrichment_Ratio	*p*_Value	Corrected_*p*	Genes	Significant
1	ECM–receptor interaction	dre04512	2	89	2.25%	0.00123	0.0135	*fras1*|*lamb4*	Yes
2	Non-homologous end-joining	dre03450	1	14	7.14%	0.00855	0.0470	*dntt*	Yes
3	Glycosphingolipid biosynthesis—lacto and neolacto series	dre00601	1	26	3.85%	0.01535	0.0563	*st3gal4*	No
4	Phagosome	dre04145	1	155	0.65%	0.08566	0.1350	*rab5ab*	No
5	Autophagy-animal	dre04140	1	162	0.62%	0.08933	0.1350	*ern2*	No
6	Apoptosis	dre04210	1	167	0.60%	0.09195	0.1350	*ern2*	No
7	Apelin signaling pathway	dre04371	1	178	0.56%	0.09768	0.1350	*tfam*	No
8	Protein processing in endoplasmic reticulum	dre04141	1	179	0.56%	0.09820	0.1350	*ern2*	No
9	Focal adhesion	dre04510	1	243	0.41%	0.13087	0.1599	*lamb4*	No
10	Endocytosis	dre04144	1	311	0.32%	0.16435	0.1808	*rab5ab*	No
11	Metabolic pathways	dre01100	1	1507	0.07%	0.58724	0.5872	*st3gal4*	No

## Data Availability

No new data were created or analyzed in this study.
